# Pressure and cold pain threshold reference values in a pain-free older adult population

**DOI:** 10.1177/20494637241276104

**Published:** 2024-10-04

**Authors:** R Waller, E Brown, J Lim, R Nadarajah, E Reardon, A Mikhailov, L Straker, D Beales

**Affiliations:** Curtin School of Allied Health, 1649 Curtin University, Perth, WA, Australia

**Keywords:** Chronic pain, musculoskeletal pain, nociceptive pain, pain, pain threshold

## Abstract

**Background:**

More sex-specific pain sensitivity normative values from population-based cohorts in pain-free older adults are required. The aims of this study were (1) to provide sex- and age-specific normative values of pressure and cold pain thresholds in older pain-free adults and (2) to examine the association of potential correlates of pain sensitivity with pain threshold values.

**Methods:**

This study investigated sex-specific pressure (lumbar spine, tibialis anterior, neck and dorsal wrist) and cold (dorsal wrist) pain threshold estimates for older pain-free adults aged 41–70 years. This cross-sectional study used participants (*n* = 212) from the Raine Study Gen1-26 year follow-up. The association of pain thresholds, with correlates including sex, test site, ethnicity, waist-hip ratio, smoking status, health-related quality of life, depression, anxiety and stress symptoms, sleep quality, socioeconomic status and physical activity levels, was examined.

**Results:**

Values for pressure and cold pain thresholds for older pain-free adults are provided, grouped by vicennium, sex and test site (pressure). Statistically significant independent correlates of increased pressure pain sensitivity were test site, ethnicity and sex. Only lower waist/hip ratio was a statistically significant, independent correlate of increased cold pain sensitivity.

**Conclusions:**

This study provides robust sex- and age-specific normative values for pressure pain threshold and cold pain threshold for an older adult pain-free population. Combined with existing values, these data provide an important resource in assisting interpretation of pain sensitivity in clinical pain disorders and provide insights into the complex association of pain sensitivity with correlates that can be used in research.

## Introduction

The absolute number and proportion of older people are increasing globally due to improvements in health care and decreasing fertility rates.^1^ Globally, common musculoskeletal conditions account for 22.3% and 16.5% of years lived with disability in 50–69 and 70+ year old people, respectively.^2^ Pain in older adults can threaten maintaining meaningful activity, socialisation, independence and life roles to being productive citizens.^3^ Persistent pain conditions in older age such as low back pain and osteoarthritis are associated with high pain impact,^2,4^ but there is a weak relationship between disability and musculoskeletal pathology reported following imaging.^5,6^ Thus, there is increased attention on factors other than pathology that underlie musculoskeletal pain disorders, such as pain sensitivity. Heightened pain sensitivity, suggested to reflect sensitised nociceptive pathways, is an important factor associated with the persistence of musculoskeletal and postoperative pain and the severity of pain and may modify the effect of common treatments.^7–13^

Meaningful interpretation of pain sensitivity data may benefit from normative values to define a range of ‘normal’ to determine whether an individual presents with augmented nociceptive processing.^14–18^ Consensus statements recommend using a QST protocol for which there are normative data available.^19^ Two clinically relevant nociceptive stimuli, which have been discussed in detail previously, that can form part of a shorter QST protocol to measure pain sensitivity and to limit participant burden in large studies are pressure pain threshold (PPT) and cold pain threshold (CPT).^18,20^ In addition, a consensus statement for a QST protocol recommended a minimum of two parameters to adequately assess both small (e.g. cold pain threshold) and large (e.g. pressure pain threshold) afferent fibre function.^19^ Large population-based cohorts limit selection bias and allow for inclusion of a broad array of potential confounders, making them suited to measuring pain sensitivity in pain-free participants.^21^ Access to robust age-sex specific values for pain sensitivity in older adults is useful to define hypersensitivity or heightened pain sensitivity and can assist phenotyping of pain mechanisms, prognosis^14–16,18,22^ and guidance of targeted care in the older adult population.^23,24^

Current literature reporting normative sex-specific pain sensitivity values lacks data in older adult, pain-free populations.^16,17,21,25–27^ Additionally, there is little consideration of independent correlates such as age, adiposity, lifestyle factors, health-related quality of life and psychosocial symptoms, as per best practice recommendations.^14,28^ Furthermore, studies reporting pain sensitivity values have included people with pain^14,21,26,29,30^ or used cohorts with small older-adult populations.^16^ Whilst Neziri^16^ investigated a broad range of correlates, limitations were present such as mixing young and middle-aged adults. Waller^18^ reported robust normative PPT and CPT values (*n* = 617) for young adult (21–24 years) and investigated correlates, but these values are likely to vary for older adults. Currently there are limited robust age-sex specific pain sensitivity values and an understanding of correlates in pain-free older adult populations, highlighting the need for further studies.^28^

Therefore, the aims of this study were (1) to report age- and sex-specific values of pressure and cold pain thresholds in older pain-free adults and (2) to examine the association between pain thresholds (pressure and cold) and correlates of ethnicity, waist/hip ratio (WHR), smoking status, health-related quality of life, psychological status, sleep quality, socioeconomic status and physical activity levels. The outcomes will help further develop population-based normative values which have been identified as a clinical and research priority.^18,28^

## Research methods

### Study population

This was a cross-sectional study using data obtained from the Raine Study (https://rainestudy.org.au/). The Raine Study is one of the most diversely characterised prospective cohort studies globally which initially enrolled 2900 pregnant women recruited at 16–20 weeks of gestation.^31^ These pregnant women and their partners are referred to as Gen1 (i.e. generation1) and the children born into the study are referred to as Gen2 (i.e. generation 2). The current study used data obtained during the Gen1-26 year follow-up (referring to the data collected on Gen1 when the index [Gen2] participants were 26 years old). Parents were re-contacted if their children were included in the Gen2-22 year follow-up. Of the 1772 eligible parents, 1098 (636 mothers and 462 fathers) participated in the Gen1-26 year follow-up. The families in this cohort have been previously reported to be widely representative of the average Western Australian population in family structure, education, employment, income and ethnicity.^31^ Ethical approval for the Gen1-26 year follow-up was obtained from the University of Western Australia (RA/4/1/7236). Specific approval for this project was obtained from Curtin University Human Research Ethics Committee (HRE2021-0409) and the Raine Study (Project Number MUS0732).

### Data collection at the Gen1-26 year follow-up

Data for the Gen1-26 year follow-up were collected using the same measures and protocols as was used at the Gen2-22 year follow-up (Waller et al., 2016) to enable intergenerational comparisons.^31^ The Gen1-26 year follow-up ran between April 2015 and June 2017 and involved 4 h of objective testing, followed by an overnight sleep study. There were 961 participants with at least one valid measure for pressure or cold pain sensitivity. For those with at least one valid pain sensitivity measure, there were 198 participants who responded ‘no pain’ to the question, ‘how would you rate your pain in the past week?’ from the Orebrö Musculoskeletal Pain Questionnaire. A further 17 were included if they had answered ‘n/a’ to the above question and answered ‘no’ to ‘have you experienced any physical pain in the past 4 weeks?’.

### Quantitative sensory testing

A limitation of using population-based cohorts is the significant participant burden and short time allocated for collecting data; therefore, the pain sensitivity measures considered most clinically relevant were collected.^11,18^ The dependent variables of this study were PPT measured at four sites on the body (wrist, leg, neck and back) and CPT measured at the wrist. Standardised protocols consistent with current best practice recommendations were used.^18^ All quantitative sensory testing (QST) measurements were taken from the right side of the body as pain thresholds have been shown to be consistent on the left and right side in people without pain.^16,17,32^ Testing for PPT and CPT was completed in the early evening to reduce variance in the influence of the circadian rhythm on tissue sensitivity.^33,34^ The order of testing was kept consistent with PPT preceding CPT testing, as the application of cold has been shown to induce temporary mechanical hyperalgesia.^35^ For both PPT and CPT, four trials were performed with a 10 s rest period between trials. The mean threshold for each participant was calculated from the last three trials. Inter-examiner and intra-examiner reliability (intraclass correlation = 0.81–0.99 and 0.92–0.95, respectively) for PPT testing by the Raine Study research staff has been demonstrated as excellent and there were acceptable levels of standard error of measurement.^18^

### Pressure pain thresholds

PPT was measured using a pressure algometer (Somedic AB, Sweden) positioned perpendicular to the skin, with a contact area of 1 cm^2^. Pressure was increased at a ramp rate of 50 kPa per second, until the participant first perceived pain detection or discomfort. There was a cut-off point of 1000 kPa for safety purposes.^36^ Instructions given to participants were, ‘The moment the pressure increases to a point where it first feels uncomfortable or painful, press and release the button. This means the very first onset of discomfort or pain and not the most pressure that you can bear’. The four PPT testing sites were the following: the central aspect of the dorsal wrist joint line (wrist); middle of tibialis anterior muscle belly, 2.5 cm lateral and 5 cm distal to the tibial tubercle (leg); upper trapezius muscle belly at the mid-point between the C7 spinous process and the lateral acromion (neck); and the erector spinae, 2 cm lateral to the L4/5 interspinous space (back). Testing was repeated four times with a 10 s rest between each test. The mean from the last three tests was used for analysis. A lower score indicates a greater degree of pressure pain sensitivity.

### Cold pain threshold

CPT was measured using an Advanced Thermo-sensory Stimulator 2, 2001 (Medoc) or a Modular Sensory Analyzer (Somedic) thermal stimulator with a thermode of 9.0 cm^2^ and 12.5 cm^2^, respectively, applied on the dorsum of the right wrist. Temperature was decreased at a rate of 1^o^C until the participant first perceived or detected pain. There was a baseline temperature of 32^o^C which decreased at a rate of 1^o^C/second to a cut-off of 5^o^C. For CPT, participants were read standardised instructions of ‘allow the temperature to drop until the moment it reaches a point where it feels uncomfortably or painfully cold, and then press the button. This means the very first onset of discomfort or pain and not the most cold that you can bear’. A higher score indicates a greater degree of cold pain sensitivity.

### Other variables

To investigate possible associations with pain sensitivity measures, several other variables were collected based on their known associations with pressure and cold pain sensitivity measures.

### Ethnicity

Race was recorded for both males and females as part of a questionnaire prior to testing. The five original categories recorded for our study of Gen1 were as follows: (1) Caucasian; (2) Chinese; (3) Vietnamese; (4) Indian; and (5) Other. Given a high proportion of Caucasian representation, the categories were dichotomised to Caucasian and non-Caucasian.

### Waist/Hip ratio

A standardised protocol was employed to measure the waist and hip circumferences using a metric tape measure, to calculate the WHR. Previous Raine Study investigation has reported an association with WHR and pain sensitivity but not with body mass index (BMI) and pain sensitivity, with WHR therefore considered as the optimal adiposity covariable.^18^

### Smoking status

Participants were asked ‘Do you currently smoke manufactured or hand-rolled cigarettes?’ and were subsequently classified as non-smokers or smokers.

### Health-related quality of life

Health-related quality of life was measured using the Short Form-12, version 2 (SF-12), a self-reported, validated and reliable questionnaire which assesses the self-rated impact of health on an individual’s quality of life.^37^ Two summary measures are produced from 12 questions: a Mental Component Summary (MCS) and a Physical Component Summary (PCS).^37^ Each SF-12 scale is a norm-based score with a mean of 50 and a standard deviation of 10, with higher scores indicating better health-related quality of life.^37^

### Psychological data

The severity of depression, anxiety and stress symptoms were measured using the Depression Anxiety Stress Scale (DASS-21), with good evidence for validity and reliability.^38^ Higher scores indicate greater symptom severity, with established cut-off scores for mild, moderate, severe and extremely severe levels of symptoms for each sub-scale.^39^

### Sleep quality

Sleep quality was measured using the Pittsburgh Sleep Quality Index (PSQI), which is considered a reliable and valid measure of sleep quality.^40^ This questionnaire assesses the self-rated perceived quality of sleep over the prior month period with higher scores indicating poorer sleep quality.^40^

### Socioeconomic status

Participants were asked, ‘What is your household gross income and/or benefit per week?’. Data was initially represented in 15 categories which were simplified into the following five categories: (1) $0–$299/week; (2) $300–$999/week; (3) $1,000–$1,999/week; (4) $2,000–3,499/week; (5) $3,500–$5,000+/week.

### Physical activity

Physical activity was measured using the validated and reliable International Physical Activity Questionnaire (IPAQ).^41^ Participants were asked the number of minutes per day that they were moderately physically active in the past week.

### Statistical analysis

To provide pressure and cold pain sensitivity values (with 95% confidence intervals) for pain hypersensitivity, quantile regression analyses were conducted to determine the 5^th^, 10^th^ and 25^th^ percentiles for PPT and 95^th^, 90^th^ and 75^th^ percentiles for CPT, estimated with bootstrapped standard errors (1000 replications).^18^ Normative values (with 95% confidence intervals) for pain hyposensitivity were determined using the 75^th^, 90^th^ and 95^th^ percentiles for PPT and 25^th^, 10^th^ and 5^th^ for CPT. For the reporting of normative values, participants were categorised by age into two vicennial groups with a moving average: 40–60 years and 50–70 years. Thus, participants with ages 50–60 years are included in both groups. Using a moving average window provided a larger sample size in each window and therefore more robust estimates.

For all participants (i.e. not by age group), multivariable independent correlates of PPT were determined with linear regression utilising generalised estimating equations, with an exchangeable correlation structure (r = 0.76) to account for the non-independence of the data due to repeated measures by test site. For all participants (i.e. not by age group), multivariable independent correlates of CPT were determined using Tobit regression, a non-parametric test appropriate to analyse the left-censored data due to the lower limit of the Somedic thermal analyser being 5°C. For both PPT and CPT, a series of univariable analyses were initially performed for sex, test site (PPT only), ethnicity, WHR, smoking, quality of life, psychological measures, sleep quality, income and activity. All univariable associations were adjusted for sex. Sex, and variables with sex-adjusted associations of *p* < .15, were entered into multivariable regression models, using a purposeful selection of covariate approach, to ensure selection of important confounding variables.^42^ In the multivariable model, covariates with significance at *p* < .05 were retained. Each model was examined to ensure absence of influential observations and collinearity of variables, and linearity of associations and normality and homoscedasticity of residuals. Stata/IC Version 15.1 for Windows (StataCorp LP, College Station, TX, USA) was used for all these analyses.

To aid interpretation of the reported pain sensitivity values, comparison with PPT and CPT values by site and sex was done with young adults of the Raine Study^18^ and the most age-relevant comparison datasets from Neziri^16^ and Magerl.^15^ The mean differences, significance value (*p*-value) and 95% confidence interval of the differences were calculated using the mean, standard deviation and sample size, respectively (MedCalc, Version 22.013).

## Results

There were 212 pain-free older adults included in our study, of which 115 were female (53.5%) and 97 male (46.5%). The mean (SD) age of participants was 57.1 (5.4) years, with a range of 40.8–69.9 years. There were three male participants greater than 70 year of age with relevant data, but they were not included in the analysis as they were classified as outliers. Further demographic information is presented in Table 1 for all participants and for both the vicennial age groups. Statistical analysis to investigate the association between age and other correlates for all participants (i.e. not by age group) showed statistical significance for sex (males were older than females), smoking (less likely to smoke with increasing age) and income (increased with age).

**Table 1.** Demographic summary statistics.

Variables	All participants *n* = 215			40–60 years *n* = 144		50–70 years *n* = 191	
	Mean (SD) Median (IQR) or N (%)	Range	Overall *p*-Value for age	Mean (SD) or N (%)	Range	Mean (SD) or N (%)	Range
Age (years)	57.1 (5.4)	40.8–69.9		54.3 (4.0)	40.8–59.9	58.2 (4.4)	50.0–69.9
Sex (female)	115 (53.5%)		<0.001	82 (56.9%)		99 (51.8%)	
Ethnicity (Caucasian)^a^	193 (91.5%)		0.118	134 (93.7%)		174 (91.6%)	
Waist/hip ratio^a^	0.91 (0.09)	0.69–1.13	0.035	0.90 (0.09)	0.70–1.13	0.91 (0.88)	0.68–1.13
Female	0.86 (0.07)	0.69–1.04	0.386	0.86 (0.07)	0.70–1.03	0.86 (0.08)	0.68–1.04
Male	0.96 (0.07)	0.79–1.13	0.994	0.96 (0.07)	0.79–1.13	0.96 (0.07)	0.79–1.13
Smoking (yes)^b^	14 (7.0%)		0.016	12 (9.3%)		13 (7.4%)	
SF-12							
PCS^c^	54.7 (5.7)	20.7–65.0	0.553	55.2 (5.2)	32.6–65.0	55.0 (5.4)	20.7–65.0
MCS^c^	52.6 (7.4)	20.9–66.3	0.163	52.0 (7.4)	22.6–65.2	52.7 (7.3)	20.9–66.3
DASS-21							
Depression^d^	2.6 (4.1)	0–28	0.617	2.6 (4.2)	0–28	2.5 (4.0)	0–28
Anxiety^d^	1.6 (2.7)	0–16	0.450	1.6 (2.6)	0–16	1.6 (2.6)	0–16
Stress^e^	4.2 (5.0)	0–20	0.493	4.1 (4.8)	0–20	4.2 (5.0)	0–20
Sleep							
PSQI score^f,^^n^	5.1 (3.2)	0–17	0.384	5.1 (3.2)	0–16	5.0 (3.1)	0–17
Socioeconomic							
Income/week^g^	3.8 (1.1)	1–5	0.011	3.7 (1.1)	1–5	3.5 (1.2)	1–5
Activity							
MPA^h^	53.9 (63.7)	0–180	0.299	59.4 (64.1)	0–180	53.8 (64.2)	0–180
PPT (kPa)							
Back^i^	518.4 (209.6)	141.7–1000	0.804	513.8 (207.7)	157.0–1000	522.3 (208.4)	141.7–1000
Leg^j^	453.33 (209.5)	97–1000	0.579	494.1 (201.0)	163.0–1000	487.5 (208.4)	97.0–1000
Neck^k^	410.3 (213.0)	91–1000	0.871	416.6 (211.2)	122.3–1000	413.0 (217.9)	91.0–1000
Wrist^l^	418.8 (174.0)	75–1000	0.781	415.9 (168.0)	131.3–1000	419.0 (169.8)	75–1000
CPT (°C)							
Wrist^m^	8.7 (6.2) 5 (5, 10)	5–29.4	0.616	8.3 (5.9)	5–28.1	8.6 (6.1)	5–29.4

Missing.

^a^ = 1.

^b^ = 11.

^c^ = 19.

^d^ = 13.

^e^ = 16.

^f^ = 6.

^g^ = 8.

^h^ = 9.

^i^ = 5.

^j^ = 3.

^k^ = 4.

^l^ = 2.

^m^ = 5.

The a-m superscript represent the number of missing values.

^n^ = income five categories: (1) $0–$299/week; (2) $300–$999/week; (3) $1,000–$1,999/week; (4) $2,000–3,499/week; (5) $3,500–$5,000+/week.

SF-12: 12-Item Short Form Health Survey; PCS: Physical Component Score; MCS: Mental Component Score; DASS-21: Depression, Anxiety, & Stress Scale (21); PSQI: Pittsburgh Sleep Quality Index; MPA: moderate physical activity (minutes/day); PPT: pressure pain threshold; CPT: cold pain threshold.

A summary of pain sensitivity values with 95% confidence intervals stratified by age group, sex and test site for PPT and by age and sex for CPT is outlined in Table 2. For PPT and CPT, values for hypersensitivity are ordered from the most sensitive to least sensitive, while values for hyposensitivity are ordered from the least insensitive to most insensitive. The 5^th^, 10^th^ and 25^th^ percentiles are listed for PPT hypersensitivity; and the 75^th^, 90^th^ and 95^th^ percentiles are listed for PPT hyposensitivity. For PPT, 3.5% and 0.7% of measurements for males and females reached the 1000 kPa limit, respectively. The 75^th^, 90^th^ and 95^th^ percentiles are listed for CPT hypersensitivity. Values for CPT hyposensitivity were unable to be estimated in both age groups as more than half of the males (52%) and females (54.8%) reached the 5^o^C minimum cut-off.

**Table 2.** Pain sensitivity values for pressure and cold pain thresholds with 95% confidence intervals stratified by age, sex and test site.

Pain threshold (PPT – kPa)	Females				Males			
	Number	Mean (SD)	Hypersensitivity	Hyposensitivity	Number	Mean (SD)	Hypersensitivity	Hyposensitivity
			p⁵ (95% CI)	p⁷⁵ (95% CI)			p⁵ (95% CI)	p⁷⁵ (95% CI)
			p^1^⁰ (95% CI)	p⁹⁰ (95% CI)			p^1^⁰ (95% CI)	p⁹⁰ (95% CI)
			p^2^⁵ (95% CI)	p⁹⁵ (95% CI)			p^2^⁵ (95% CI)	p⁹⁵ (95% CI)
PPT back								
40–60 years	79	468 (185)	228 (203, 274), 268 (218, 299), 330 (295, 378)	567 (495, 638), 744 (635, 878), 856 (715, 934)	61	574 (221)	228 (175, 338), 293 (212, 391), 427 (354, 484)	728 (627, 809), 897 (781, 1000), 961 (853, 1000)
50–70 years	96	465 (172)	217 (179, 273), 264 (212, 300), 333 (300, 384)	576 (495, 620), 723 (624, 820), 843 (690, 902)	91	583 (227)	222 (157, 315), 294 (217, 374), 424 (362, 468)	738 (675, 812), 914 (812, 983), 975 (923, 1000)
PPT leg								
40–60 years	80	440 (166)	206 (165, 252), 249 (193, 285), 331 (284, 367)	523 (469, 599), 662 (594, 754), 753 (617, 884)	62	563 (222)	262 (223, 312), 297 (247, 340), 383(313, 446)	715 (642, 823), 883 (733, 1000), 962 (823, 1000)
50–70 years	97	416 (164)	178 (147, 217), 219 (168, 271), 298 (271, 340)	518 (460, 555), 661 (555, 723), 764 (617, 807)	92	563 (223)	269 (223, 300), 303 (259, 336), 382 (333, 429)	692 (644, 825), 909 (823, 1000), 985 (891, 1000)
PPT neck								
40–60 years	80	360 (159)	155 (126, 205), 189 (150, 222), 244 (222, 274)	451 (374, 538), 623 (507, 694), 680 (631, 714)	61	491 (246)	174 (138, 225), 209 (151, 258), 292 (231, 362)	658 (548, 782), 875 (683, 960), 940 (864, 980)
50–70 years	97	348 (163)	138 (96, 169), 169 (129, 207), 236 (207, 260)	435 (379, 519), 612 (530, 674), 679 (631, 759)	91	482 (247)	176 (150, 208), 205 (172, 247), 275 (243, 325)	636 (579, 742), 868 (742, 957, 953 (868, 1000)
PPT wrist								
40–60 years	80	368 (150)	183 (148, 215), 213 (182, 246), 269 (241, 294)	444 (386, 534), 566 (532, 613), 522 (550, 794)	62	477 (171)	232 (161, 275), 266 (231, 310), 355 (291, 422)	580 (529, 632), 673 (592, 803), 759 (643, 894)
50–70 years	98	356 (124)	176 (161, 209), 208 (173, 240), 269 (240, 295)	453 (393, 520), 555 (520, 573), 593 (543, 613)	92	486 (187)	228 (161, 265), 263 (238, 302), 341 (300, 403)	576 (534, 643), 714 603, 885), 839 (732, 995)

Pain threshold (CPT – °C)	Females				Males			
	Number	Mean (SD)	Hypersensitivity	Hyposensitivity	Number	Mean (SD)	Hypersensitivity	Hyposensitivity
			p⁹⁵ (95% CI)	p^2^⁵ (95% CI)			p⁹⁵ (95% CI)	p^2^⁵ (95% CI)
			p⁹⁰ (95% CI)	p^1^⁰ (95% CI)			p⁹⁰ (95% CI)	p^1^⁰ (95% CI)
			p⁷⁵ (95% CI)	p⁵ (95% CI)			p⁷⁵ (95% CI)	p⁵ (95% CI)
CPT wrist								
40–60 years	80	8.4 (6.0)	22.5 (16.9, 26.4), 18.2 (12.8, 22.5), 9.6 (5.6, 13.3)	Not estimated	59	8.3 (6.0)	22.5 (16.8, 27.1), 18.4 (12.7, 23.4), 9.4 (5.5, 14.7)	Not estimated
50–70 years	97	8.4 (6.0)	22.7 (17.0, 28.1), 18.4 (12.9, 22.1), 9.6 (5.6, 12.9)	Not estimated	90	9.0 (6.5)	23.8 (19.8, 25.0), 20.6 (15.8, 23.4), 10.0 (6.9, 16.1)	Not estimated

PPT = pressure pain threshold; p⁵ = 5^th^ percentile; p^1^⁰ = 10^th^ percentile; p^2^⁵ = 25^th^ percentile; p⁷⁵ = 75^th^ percentile; p⁹⁰ = 90^th^ percentile; p⁹⁵ = 95^th^ percentile; CI = confidence interval. PPT values for hypersensitivity are ordered from the most sensitive to least sensitive, while values for hyposensitivity are ordered from the least insensitive to most insensitive.

CPT = cold pain threshold; p⁵ = 5^th^ percentile; p^1^⁰ = 10^th^ percentile; p^2^⁵ = 25^th^ percentile; p⁷⁵ = 75^th^ percentile; p⁹⁰ = 90^th^ percentile; p⁹⁵ = 95^th^ percentile; CI = confidence interval. CPT values for hypersensitivity are ordered from the most sensitive to least sensitive, while values for hyposensitivity are ordered from the least insensitive to most insensitive.

Due to the non-significant association of age and pain sensitivity (PPT and CPT) in this study, univariable regression analysis of pain sensitivity and correlates were calculated without subgrouping by age (Table 3). For PPT, there was statistical significance (*p* < .001) for decreased pressure pain sensitivity of the back compared with leg, neck and wrist PPT test sites, and of the leg compared to the neck and wrist test sites. There was no statistically significant difference for sensitivity between neck and wrist test sites. Females were more sensitive to pressure than males (mean difference = −120.9 kPa, *p* < .001; 95% CI: −167.4, −74.3). Across all age groups, most participants reported a normal range for DASS-21 scores at 87.4%, 89.8% and 87.4% for depression, anxiety and stress symptoms, respectively. In the multivariable model analysis, only sex and test site remained significant for PPT, meaning the normative pain sensitivity values reported in Table 2 did not change. For CPT values, there was no statistical significance between males and females. In females, a higher WHR was associated with less sensitivity to cold (mean difference = −3.1˚C, p= .045, 95% CI: −6.3, −0.1). No other covariables showed statistically significant associations for CPT regression analysis. In the multivariable model analysis for CPT, there were no significant findings, meaning the normative pain sensitivity values reported in Table 2 did not change.

**Table 3.** Univariable regression model for PPT (kPa) and CPT (°C) including all participants.

Variables	Regression coefficient PPT (95% CI)^a^	*p*-Value^a^	Regression coefficient CPT (95% CI)^b^	*p*-Value^b^
Age	−2.5 (−6.9, 2.0)	0.277	0.1 (−0.3, 0.4)	0.701
Sex (male)	Ref			
Female	−129.4 (−177.3, −81.6)	<0.001	−1.0 (−4.5, 2.6)	0.593
Race (Caucasian)	Ref			
Other	83.0 (−0.4, 166.5)	0.051	0.5 (−5.6, 6.6)	0.864
Waist-hip ratio^c^	1.9 (−1.4, 5.2)	0.255	−2.6 (−5.0, −0.2)	0.037
Male	20.7 (−36.1, 77.5)	0.475	−2.1 (−5.7, 2.1)	0.354
Female	17.6 (−19.4, 54.6)	0.351	−3.1 (−6.3, −0.1)	0.045
Smoking (no)	Ref			
Yes	54.0 (−39.4, 147.4)	0.257	1.4 (−5.7, 8.5)	0.704
SF 12				
PCS	0.3 (−4.2, 4.9)	0.882	−0.2 (−0.5, 0.1)	0.293
MCS	0.4 (−3.0, 3.8)	0.780	−0.0 (−0.3, 0.2)	0.802
DASS				
Depression	−4.7 (−10.8, 1.4)	0.127	0.4 (−0.1, 0.9)	0.092
Anxiety	−4.4 (−14.1, 5.4)	0.378	0.3 (−0.4, 0.9)	0.447
Stress	−4.1 (−9.2, 0.8)	0.106	0.3 (−0.1, 0.6)	0.154
Sleep (PSQI score)	2.6 (−5.0, 10.1)	0.502	−0.3 (−0.8, 0.3)	0.381
Socioeconomic				
Income/week^d^	−9.6 (−31.3, 12.1)	0.384	−0.5 (−2.0, 1.1)	0.564
Activity (MPA)	0.3 (−0.1, 0.7)	0.116	−0.01 (−0.04, 0.02)	0.530
Site				
Leg vs back	−31.3 (−49.7, −12.9)	0.001	**—**	**—**
Neck vs back	−107.9 (−126.3, −89.5)	<0.001	**—**	**—**
Wrist vs back	−99.5 (−117.9, −81.1)	<0.001	**—**	**—**
Neck vs leg	−76.6 (−94.9, −58.2)	<0.001	**—**	**—**
Wrist vs leg	−68.1 (−86.5, −49.8)	<0.001	**—**	**—**
Wrist vs Neck	8.4 (−9.9, 26.8)	0.368	**—**	**—**

^a^ = univariable regression with generalised estimating equations, adjusted for sex.

^b^ = non-linear Tobit regression, left censored, adjusted for sex.

^c^ = coefficient representing the expected change for a 0.1 change in waist/hip ratio.

^d^ = income five categories: (1) $0−$299/week; (2) $300−$999/week; (3) $1,000−$1,999/week; (4) $2,000−3,499/week; (5) $3,500−$5,000+/week.

CI: confidence interval; SF-12: 12-Item Short Form Health Survey; PCS: Physical Component Score; MCS: Mental Component Score; DASS-21: Depression, Anxiety, & Stress Scale (21); PSQI: Pittsburgh Sleep Quality Index; MPA: moderate physical activity (minutes/day); PPT: pressure pain threshold; CPT: cold pain threshold.

Comparisons with existing PPT and CPT values in young adults (Gen2) of the Raine Study^18^ and the most relevant comparison datasets from Neziri^16^ and Magerl^15^ are presented in Table 4. Young adult (Gen2: 21.0–24.4 years) compared with older adult (Gen1: 50–70 years) Raine Study participants were more pressure sensitive at the back (females only: 95% CI: −124, −42; *p* = .0001) and neck (females: 95% CI: −134, −72; *p* < .0001, males: 95% CI: −178, −80; *p* < .0001) but not leg and wrist. Neziri participants (50–80 years) compared to older (50–70 years) Raine Study participants were more pressure pain sensitive at the back (females: 95% CI: −68, −156; *p* < .0001, males: 95% CI: −251, −137, *p* < .0001) and neck (males only: 95% CI: −187, −67, *p* < .0001). Young compared with older Raine Study participants were more cold pain sensitive at the wrist (females: 95% CI: 3.5, 7.1; *p* < .0001, males: 95% CI: 0.1, 3.5; *p* < .0346). Although Magerl^15^ reported the age and sex of the overall sample of 180 participants (17 to 75 years [38.4 ± 12.9], 61% female), the specific number of observations used for estimation of sex and decade specific norms was not reported, meaning a group mean comparison by age category was not able to be done.

**Table 4.** Pressure and cold pain threshold value comparison with existing data.

Pain threshold	Females				Males			
	Number	Mean (SD) or (95%CI)	Hypersensitivity (PPT: p⁵, p^1^⁰, p^2^⁵; CPT: p⁹⁵, p⁹⁰, p⁷⁵)	Hyposensitivity (PPT: p⁷⁵, p⁹⁰, p⁹⁵; CPT: p^2^⁵, p^1^⁰, p⁵)	Number	Mean (SD) or (95%CI)	Hypersensitivity (PPT: p⁵, p^1^⁰, p^2^⁵; CPT: p⁹⁵, p⁹⁰, p⁷⁵)	Hyposensitivity (PPT: p⁷⁵, p⁹⁰, p⁹⁵; CPT: p^2^⁵, p^1^⁰, p⁵)
PPT back								
Waller^a^	96	465 (172)*^#^	217, 264, 333	576, 723, 843	91	583 (227)^#^	222, 294, 424	738, 914, 975
Waller^b^	275	382 (179)*	135	726	334	541 (241)	194	999
Neziri^c^	75	353 (98)^#^	194	505	75	389 (116)^#^	259	538
PPT leg								
Waller^a^	97	416 (177)	178, 219, 298	518, 661, 764	92	563 (223)	269, 303, 382	702, 909, 985
Waller^b^	277	394 (189)	156	779	334	520 (234)	218	998
PPT neck								
Waller^a^	97	348 (163)*	138, 170, 236	435, 612, 679	91	482 (247)*^#^	176, 205, 275	636, 868, 953
Waller^b^	277	245 (121)*	92	488	334	353 (200)*	125	793
Neziri^c^	75	314 (80)	199	451	75	355 (105)^#^	223	544
PPT wrist								
Waller^a^	98	356 (124)	176, 208, 269	453, 555, 593	92	486 (187)	228, 263, 341	576, 714, 839
Waller^b^	276	360 (144)	162	942	336	492 (217)	197	940
Magerl^d^	Not reported	469 (294–751)	Not reported	Not reported	Not reported	486 (264–896)	Not reported	Not reported
CPT wrist								
Waller^a^	97	8.4 (6.0)*	22.7, 18.4, 9.6	Not estimated	90	9.0 (6.5)*	23.8, 20.6, 10.0	Not estimated
Waller^b^	274	13.7 (8.3)*	26.9	5.0	328	10.8 (7.3)*	25.7	Not estimated
Magerl^d^	90^e^	10.7 (7.9)	Not reported	Not reported	90^e^	6.5 (6.6)	Not reported	Not reported

^a^ Age range 50–70 (current study).

^b^ Age range 21.0–24.4 (R. Waller et al., 2016).

^c^ Age range 50–80 (Neziri et al., 2011).

^d^ Age range 50–60 (Magerl, Krumova et al. 2010).

^e^ Number of all female or male subjects aged 15–75, decade 50–60 years: calculated from subjects between >45 and 65 years of age (Magerl et al., 2010).

*Waller^a^ more pain sensitive than Waller^b^, *p* < .05.

^#^Neziri^c^ more pain sensitive than Waller^a^, *p* < .05.

PPT = pressure pain threshold (kPa); CPT = cold pain threshold (^o^C); p⁵ = 5^th^ percentile; p^1^⁰ = 10^th^ percentile; p^2^⁵ = 25^th^ percentile; p⁷⁵ = 75^th^ percentile; p⁹⁰ = 90^th^ percentile; p⁹⁵ = 95^th^ percentile.

## Discussion

This study provides normative pain sensitivity values for pressure and cold pain thresholds from an older-adult population in two vicennia that had been pain-free for at least 1 week prior to testing, adding to existing data. Strengths of this study are that, to date, it is amongst the largest pain-free older adult population-based cohorts reporting values for PPT and CPT data and there is consideration of multiple potential covariables. In addition, the bootstrapping model selection provides confidence in the stability of the results and estimate bounds of the 95% confidence intervals.^43^ These results when compared to previous studies that allow comparison, suggest some generalisability of results and combined these data to provide an important resource for comparison with pain populations.

### Pressure pain threshold variation with sex and age

**Figure 1.**
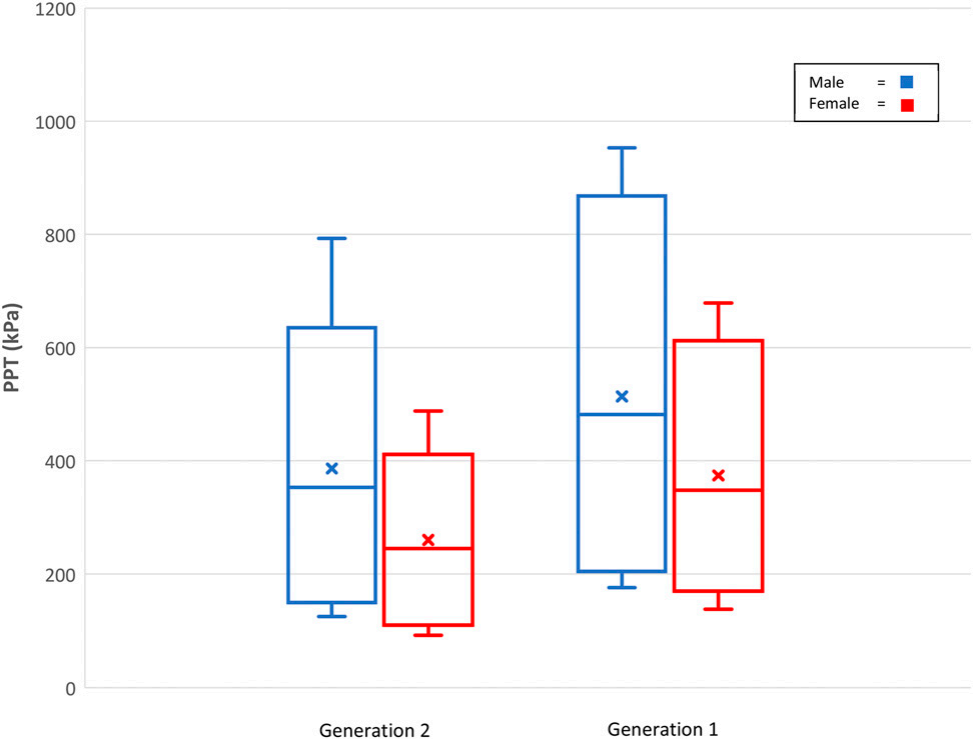
Distribution of pressure pain thresholds (kPa) at the neck for Raine Study Generation 2 and Generation 1 participants (Whiskers = 95^th^ and 5^th^ percentiles, Box = 75^th^ and 25^th^ percentiles, horizontal line = median, x = mean).

This study provides further evidence to support that females are consistently more pressure pain sensitive than males across the lifespan and pressure pain sensitivity has a tendency to decrease with age.^28^ With regards to sex differences, this study (Gen1) and the younger Raine Study adults (Gen2) both report females are more pressure sensitive than males across all reported age groups (see Figure 1 for a visual comparison). Another study reported pain-free pain sensitivity values for PPT for the back and neck by two age groups of younger (20–49 years) and older (50–80 years) Swiss adults (*n* = 300; 49% females) (Table 4).^16^ While reporting females were more pressure pain sensitive compared to men (*p* < .001), this sex difference for PPT was not statistically reported for each age category. Previously, the heritability of pressure pain sensitivity in Raine Study parents (Gen1) and their offspring (Gen2) has been reported revealing a significant genetic influence on pressure pain sensitivity.^10^ The stability of the sex difference in pressure pain sensitivity over time is probably influenced by its genetic influences, also known as heritability.

While there was no association with age and PPT within the Gen1 (50–70 years)participants, they were less pressure pain sensitive than Gen2 (20–24 years) participants at the lumbar spine (females) and the neck (females and males) but not the leg and wrist test sites. These comparisons support systematic review findings that there is tentative evidence that pressure sensitivity decreases with older age in healthy humans.^28^ In comparison, Neziri study participants (50–80 years) were significantly more pressure sensitive than Gen1 participants of equivalent age at the lumbar spine (females and males) and neck (males). Participants from both these studies were pain free, with Neziri recruiting via media advertising as opposed to Raine Study participants who are part of a population-based cohort. The different age equivalent findings might represent variation on definition of ‘pain-free’ or ‘healthy’, selection bias, relatively small sample sizes, ethnicity or geographical differences.^44^ These results support the need for age, sex and site-specific pressure pain threshold data with a future focus on adolescents and 25–50 years of age, larger sample sizes and understanding variation between populations in different geographical locations.^18,45^

### Cold pain threshold variation with sex and age

**Figure 2.**
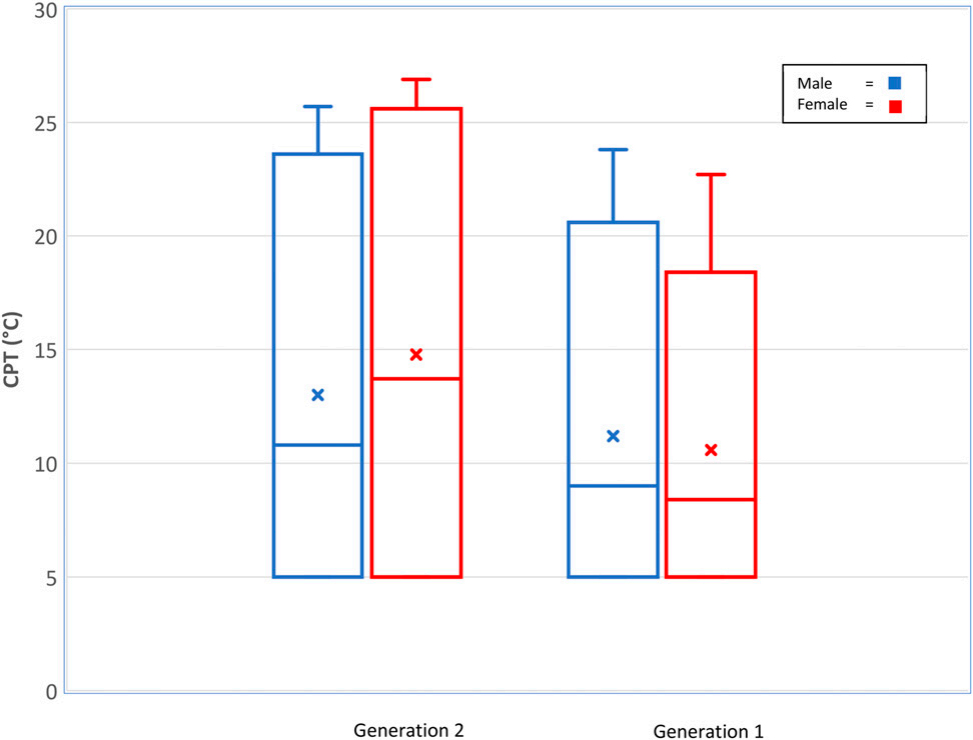
Distribution of wrist cold pain thresholds (degrees Celsius) for Raine Study Generation 2 and Generation 1 participants (Whiskers = 95^th^ percentiles, Box = 75^th^ and 25^th^ percentiles, horizontal line = median, x = mean).

This study provides evidence to support that the sex difference in cold pain sensitivity changes across the lifespan and cold pain sensitivity decreases with age (see Figure 2 for a visual comparison). However, there was no sex difference reported for cold pain sensitivity in older adult Raine Study participants. This contrasts with the younger Gen2 values,^18^ where a statistically and clinically meaningful increase in cold pain sensitivity for females was reported. While Magerl reported females were more cold pain sensitive at the hand than males in each decade between 20 and 70, the finding was not statistically significant potentially reflecting the relatively small participant numbers of 90 in each sex group.^15^

The sex and age differences in CPT changing between young and older adulthood may reflect the non-heritable nature of cold sensitivity reported in the Raine cohort implying environmental factors influence changes in CPT over time.^18^ While there is age- and sex-specific data on CPT values, there is currently no longitudinal pain sensitivity data in individuals limiting the understanding on why changes might occur in people over time with Raine Study data currently limited to young and older adults.^11^ Associations have been reported of heightened cold pain sensitivity with heightened pain experience,^20,46^ early life stress^47^ and altered hypothalamic–pituitary–adrenal axis function^48^ suggesting heightened cold pain sensitivity is associated with changes in the stress response system.^10^ Understanding why cold pain sensitivity changes over time in individuals, while currently unknown, may provide important insights into what and how environmental factors influence change in cold pain sensitivity over time and whether these factors are potential modifiers of musculoskeletal pain and targets for care.^10^

There were no statistically significant associations between cold pain sensitivity and Gen1 age in our study. Regarding age differences, Gen1 (50–70 years) were significantly less cold sensitive at the wrist than Gen2 (20–24 years), with females exhibiting a larger mean difference (5.3˚C, 95% CI: 3.5, 7.1) compared with the male participant mean difference (1.8, 95% CI: 0.1, 3.5) (Table 4). The larger mean difference of cold pain sensitivity in females with age potentially reflects changing hormonal profiles with age, specifically in peri-menopausal and post-menopausal women,^49^ and psychosocial influences.^47^ A confounding factor in this study was there was no control for the use of hormone replacement medication.

### Association of covariables

#### Cold pain threshold variation with waist/hip ratio

Our study reported that a higher WHR in females is associated with less cold pain sensitivity and the same was found in the Gen2 population.^18^ Our study population had an average WHR (0.91) that placed them in the higher health risk category for both females and males.^50^ This may indicate that increased central adiposity may reduce cold pain sensitivity at the wrist. A study by Price^51^ found that increased central adiposity resulted in less cold pain sensitivity in the abdomen; however, this was not reflected in cold pain sensitivity of the hand. The association might be peripherally mediated due to altered cold conduction in the adipose tissue at the dorsal wrist test site.

#### Other covariables

Although we found statistical significance for Caucasians being more pressure pain sensitive, this difference did not remain significant in the multivariable model. Interpretation of the pain sensitivity values needs to consider that there was a high percentage of Caucasian participants (91.5%) in the Gen2 sample, meaning there was low power to detect association, and Caucasians have previously been reported to be less sensitive than other groups such as African Americans.^52^ Smoking, sleep quality and socioeconomic status were also not statistically significantly associated with pain sensitivity. Interestingly, Waller^18^ reported a statistically significant association between smoking and decreased cold pain sensitivity in younger adults. Nicotine consumption has been demonstrated to result in an analgesic effect, decreasing pain sensitivity.^53^ The smoking rates (7.6%) of Gen1 were very low compared with Gen2 (14.7%), subsequently there was low power to detect a significant difference.^18^ There was also no significant association with activity levels and pressure or cold pain sensitivity in Gen1 which is in accordance with the Gen2 younger population.^18^

### Limitations

A limitation to this study is while pain-free has been defined as no current musculoskeletal pain (in at least the last week) and is consistent with criteria used in other studies,^15,16^ many older adults present with a range of chronic health conditions, such as comorbidity associated with neuropathy, and a history of prior pain.^23^ These influences are not accounted for and limit how data can be used in this older age group to understand the development of pain. As the Raine Study is a multigenerational study, the participants are related to the participants of the Gen2 study,^18^ and as PPT has been identified as having a genetic association,^10^ this may impact the external validity of the PPT results in this study. In addition, by the design of the Raine Study, all Gen1 females recruited were parous which may impact the generalisability of findings to nulliparous females. The CPT 5^o^C cut-off imposed due to the limitations of testing equipment was reached by more than half the participants, meaning we could not provide true estimates for hyposensitivity to cold. This may not be important where hypersensitivity to cold is of more interest clinically and in research.^54^ Furthermore, Tobit regression analysis used here does account for the censored data. There were two types of thermal stimulators used with different thermode head sizes (9 cm^2^ and 12 cm^2^). However, the type of stimulator was not recorded at testing which did not allow sensitivity testing for an effect of head size and there remains the possibility this influenced results.

## Conclusion

In conclusion, this study adds to existing sex- and age-specific pain sensitivity data for pressure pain threshold tested at four body sites and cold pain threshold at the dorsal wrist in older adults aged 41–70 years. The data from a large population-based, non-clinical cohort based in a Mediterranean climate limits selection bias and allowed investigation of a broad number of potential correlates. These data add to existing resources, enhancing the ability to more accurately phenotype clinical pain disorders in older adults.
